# The protocol of Enhanced Recovery After Cardiac Surgery (ERACS) in congenital heart disease: a stepped wedge cluster randomized trial

**DOI:** 10.1186/s12887-023-04422-2

**Published:** 2024-01-05

**Authors:** Dou Dou, Yuan Jia, Su Yuan, Yang Wang, Yinan Li, Hongbai Wang, Jie Ding, Xie Wu, Dongyun Bie, Qiao Liu, Ran An, Haoqi Yan, Fuxia Yan

**Affiliations:** 1 Department of Anesthesiology, State Key Laboratory of Cardiovascular Disease, Fuwai Hospital, National Center for Cardiovascular Diseases, Chinese Academy of Medical Sciences & Peking Union Medical College, No. 167 BeilishiRd, Xicheng District, Beijing, 100037 China; 2 Department of Medical Research & Biometrics Center, State Key Laboratory of Cardiovascular Disease, Fuwai Hospital, National Center for Cardiovascular Diseases, Chinese Academy of Medical Sciences & Peking Union Medical College, No. 167 BeilishiRd, Xicheng District, Beijing, 100037 China

**Keywords:** Enhanced recovery after surgery, Congenital disease, Major adverse cardiac and cerebrovascular events, Postoperative pulmonary complications, Acute kidney injury

## Abstract

**Background:**

The Enhanced Recovery After Cardiac Surgery (ERACS) programs are comprehensive multidisciplinary interventions to improve patients’ recovery. The application of the ERAS principle in pediatric patients has not been identified completely.

**Methods:**

This study is a multicenter, stepwise design, cluster randomized controlled trial. 3030 patients presenting during control and intervention periods are eligible if they are aged from 28 days to 6 years old and awaiting elective correction surgery of congenital heart disease with cardiopulmonary bypass. 5 centers are randomly assigned to staggered start dates for one-way crossover from the control phase to the intervention phase. In the intervention periods, patients will receive a bundle strategy including preoperative, intraoperative, and postoperative approaches. During the control phase, patients receive the usual care. The primary outcome consists of major adverse cardiac and cerebrovascular events (MACCEs), postoperative pulmonary complications (PPCs), and acute kidney injury (AKI).

**Discussion:**

This study aims to explore whether the bundle of ERAS measurements could improve patients’ recovery in congenital heart surgery.

**Trial registration:**

http://www.clinicaltrials.gov. (NCT05914103).

**Supplementary Information:**

The online version contains supplementary material available at 10.1186/s12887-023-04422-2.

## Introduction

Enhanced Recovery After Surgery (ERAS) strategy aims to improve the patient’s postoperative recovery by several evidence-based measurements. With the advantages of ERACS in adult, the application of ERACS in pediatric patients has been improved [1].

Previous studies have shown that several measurements can be applied into pediatric patients to improve the quality of postoperative recovery [2, 3]. Multiple clinical studies on pediatric ‘fast-track’ protocols have shown that these approaches can be effectively and safely applied into pediatric cardiac anesthesia without increasing adverse event rates [4–6].

However, the level of relevant evidence remains unclear. And clinical research concerning cardiac surgery primarily consists of retrospective studies, which lack prospective, large-scale, and multicenter RCT investigations. Therefore, this study aims to conduct a prospective randomized controlled trial to compare the application of the ERAS management protocol and traditional management protocol in congenital heart surgery under extracorporeal circulation. We assume that compared to traditional management schemes, ERAS management schemes can reduce the incidence of major adverse events in patients after surgery, decrease hospital stay, ICU time, and extubation time, improve patient prognosis, and accelerate postoperative recovery.

## Methods

This study employs a multicenter, stepwise design for a cluster randomized controlled trial. The scheme primarily includes preoperative, intraoperative, and postoperative phases, applied to pediatric cardiac surgery under cardiopulmonary bypass (CPB). The main objective of the study is to explore whether the ERAS management strategy can reduce the incidence of major adverse events (such as major cardiovascular and cerebrovascular complications, major pulmonary complications, and acute kidney injury).

The secondary research objective is to examine whether the ERAS management strategy can decrease patient hospitalization time, ICU time, extubation time, drainage tube removal time, hospitalization costs, pain scores, opioid drug use, and the incidence of other adverse events (including reintubation, reoperation, delirium, infection, etc.). Furthermore, the study aims to compare patients’ prognosis and outcomes compared to traditional measurements.

### Patients

The patient inclusion criteria are as follows: (1) male or female pediatric patients aged 28 days to 6 years, and (2) patients awaiting elective correction of congenital heart disease with cardiopulmonary bypass.

Patient exclusion criteria are as follows: (1) risk adjustment for congenital heart surgery (RACHS) class above class V, (2) cardiac assist device, mechanical ventilation or the history of asphyxia, (3) pulmonary disease, including respiratory tract infections and asthma, (4) severe liver or renal dysfunction, including severe acute or chronic renal dysfunction need renal replacement therapy, and acute or chronic liver failure need artificial liver therapy, (5) fetal malformation includes polysplenia syndrome, asplenia syndrome, Down syndrome, DiGeoge syndrome, Marfan syndrome, trachea-bronchus stricture, diabetes, nervous functioning disorders, genital system malformations, imperforate anus, and Williams syndrome, (6) current enrollment in another clinical trial, and (7) guardian’s refusal or low adherence.

**Fig. 1:**
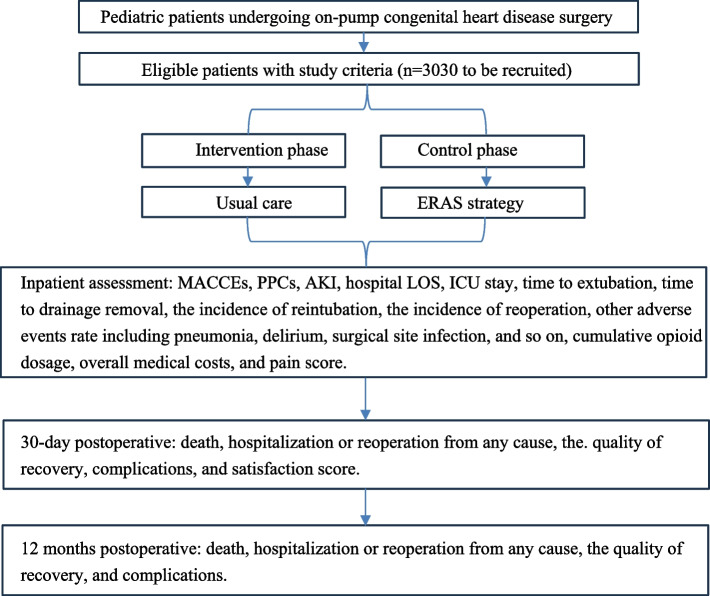
Study flowchart

 All guardians must be willing to provide informed consent to participate in the study and may withdraw from the study at any time upon their own request (Fig. 1).

### Interventions

ERAS is a complex intervention strategy trial that consists of multiple phases and components. A bundle of evidence-based perioperative strategies can be divided into preoperative, intraoperative, and postoperative phases (Appendix 1).

#### Preoperative strategies

The preoperative phase consists of education and counseling, sedation, and preoperative oral intake of multidimensional carbohydrate beverages.

Patient education and counseling are recommended to be completed by surgeons, nurses, and anesthesiologists, respectively. Guardians are suggested to be educated for 15 to 20 min. These efforts should include explanations of procedures and goals that may help reduce postoperative complications and enhance recovery.

Preoperative oral intake of multidimensional carbohydrate beverages refers to the oral administration 2 to 5 ml·kg^−1^ of energy clear beverages to pediatric patients 2 to 4 h before surgery.

The measurement of sedation recommends parients’ company and intranasal administration of dexmedetomidine 1 to 2 µg·kg^−1^ or oral administration midazolam 0.5 mg·kg^−1^.

#### Intraoperative strategies

The intraoperative phase consists of continuous infusion of dexmedetomidine, multimodal analgesia, blood conservation, prevention of postoperative nausea and vomiting, and normothermia.

Continuous infusion of dexmedetomidine is suggested to inject dexmedetomidine intravenously at the rate of 0.2~0.7 µg·kg^−1^·h^−1^.

Multimodal analgesia aims to decrease perioperative opioid consumption. We recommend anesthesiologists to select at least one of the following methods, including local anesthesia around the incision, or ultrasound-guided nerve block before incision or upon leaving the operating room.

Blood conservation typically focuses on using tranexamic acid, cell saver, and use of modified ultrafiltration to reduce blood cell transfusions.

Prevention of postoperative nausea and vomiting suggests supplying 0.15 mg·kg^−1^ dexamethasone or 0.1 mg·kg^−1^ ondansetron before anesthesia (maximum dose not exceeding 4 mg).

#### Postoperative strategies

The postoperative phase consists of early extubation, multimodal analgesia, goal-directed fluid therapy, and early removal of lines, tubes, and wires.

Extubation immediately after surgery or within 6 h of surgery is recommended for pediatric patients who meet extubation criteria. Early extubation can be achieved through time-directed extubation protocols and low-dose opioid anesthesia.

Multimodal analgesia recommends acetaminophen or non-steroidal anti-inflammatory drugs (NSAIDs). Patients would be advised to oral application of acetaminophen 4 times daily, with a total daily dose of 30 mg/kg. Ketorolac would be recommended to injected intravenously as needed, with a single dose of 0.5 to 1 mg·kg^−1^.

Goal-directed fluid therapy employs monitoring techniques to guide clinicians in administering fluids, vasopressors, and inotropes to prevent hypotension and low cardiac output. Quantified goals include blood pressure, cardiac index, systemic venous oxygen saturation, and so on.

Early feeding and physical exercise protocols suggest that drinking can be attempted 2 h after extubation and sitting or exercising can be attempted 4 h after extubation.

### Implementation of interventions

Our team will employ a multi-component implementation strategy to complex these interventions. Each intervention site will assemble an implementation team and members will receive coaching and be taught one month before the intervention phase. An accuracy rate of testing over 80% in local teams will be authorized to start intervention. Meanwhile, main implementation team personnel will collaborate with each local implementation team in a formative evaluation to identify barriers and facilitators to the implementation of the clinical intervention. The central teamworkers also monitor implementation progress and adjust strategies as necessary. The implementation outcomes will encompass metrics related to the quantity, quality, timing, and duration of implementation strategies, as well as participant and staff responsiveness, and medical record-keeping.

### Outcomes

The primary outcome is the rate of the composite outcome. The primary outcome is defined as the time-to-event of a composite of major adverse cardiac and cerebrovascular events (MACCEs), postoperative pulmonary complications (PPCs), and acute kidney injury (AKI) (Table 1).

**Table 1:** Primary outcome

Complication	Content	Definition
MACCEs	all-cause death	death for any reason
	LCOS	VIS score above 20 points
	postoperative mechanical circulatory support	ECMO, VAD, and so on
PPCs	moderate to severe atelectasis	more than 2 lobes of atelectasis diagnosed by computed tomography (CT)
	lung injury	oxygen index (PaO_2_/FiO_2_) less than 100
AKI	moderate to severe AKI	KDIGO stage 2 to 3

MACCEs include all-cause death, early postoperative low cardiac output syndrome (LCOS), and the application of postoperative mechanical circulatory support. LCOS is defined as the vasoactive inotropic score (VIS) score above 20 points. Postoperative mechanical circulatory support include extracorporeal membrane oxygenation (ECMO), ventricular assist device (VAD), and so on.

PPCs include moderate to severe atelectasis and lung injury. Moderate to severe atelectasis is defined as more than 2 lobes of atelectasis diagnosed by computed tomography (CT). Lung injury is defined as an oxygen index (PaO_2_/FiO_2_) less than 100.

AKI is defined as Kidney Disease Improving Global Outcomes (KDIGO) stage 2 to 3. We use the most proximal preoperative creatinine concentration as the baseline and the highest recorded postoperative creatinine concentration within hospitalization as the postoperative concentration.

The secondary outcomes include the following measures: hospital length of stay (LOS), ICU stay duration, time to extubation, time to drainage removal, the incidence of reintubation, the incidence of reoperation, and other adverse event rates such as pneumonia, delirium, surgical site infection, and more. Additionally, we will assess cumulative opioid dosage, overall medical costs, the numeric rating scale, and 30-day and 1-year follow-up endpoints after discharge.

These follow-up endpoints comprise mortality, postoperative hospitalization or reoperation for any reason, quality of recovery, complications, and satisfaction scores.

### Study design

We will employ a cross-sectional stepped-wedge cluster randomized design with 5 sequences and 6 periods. Each step’s length will be 2 months in duration. The unit of randomization will be the hospital site, as the trial evaluates a hospital-level intervention that requires involvement of multiple units within the hospital. We chose a stepped-wedge design because it allows all sites to eventually receive the intervention. The staggering of trial initiation at different sites also simplifies the logistical complexity of the trial.

In the first period, no hospital has access to the ERAS, and detailed measurements will not be open at any site. One month in advance of the allocated time of crossing-over, each site will be given notice of their randomization date so that they can prepare for the implementation of the ERAS strategies. A site will officially launch into the active intervention arm only on the date of randomization. After crossing over to the active intervention arm, the ERAS will become available to the hospital at the onset of each sequence.

### Randomization

The included hospitals are randomly assigned to a sequence that indicates the period during which crossover from the control phase (group C) to the intervention phase (group I) would occur. The allocation is carried out by an independent statistician. Allocations are concealed from the sites, investigators, and research staff until 1 month before the allocated start time for the relevant hospitals (Appendix 2).

### Sample power

For the trial, power calculations were performed based on analysis of medical records data from Fuwai Hospital and studies reported previously, revealing that the rate of the primary composite outcomes was 24%. We would consider a 8% relative reduction in the control group. A stepped-wedge design with 5 centers and an average of 101 patients per cluster-period (a total of 3030 patients over 6 periods in total, combining the control and intervention periods) has 90% power to detect difference and using a two-sided test at the 5% level of significance. Our calculation assumes an intracluster correlation coefficient of 0.01. No adjustment is made for cluster attrition as the risk of attrition is extremely low.

### Statistical analysis

Demographic data and baseline characteristics will be summarized as numerical means (standard deviation) or median (minimum and maximum) for continuous variables and as the number of patients (percentage) for categorical ones. The analyses for the primary composite outcomes between the two groups will be conducted using the χ2 test. Additional analyses with the Student t-test (for continuous variables) and the χ2 test (for categorical variables) will be employed for the secondary endpoints. Multiple imputation will be performed for the primary outcome in case of missing data, with single imputation performed as a sensitivity analysis.

The statistical analysis will be based on the full analysis set (FAS) and per protocol set (PPS). We will use completed cases with more than 8 items of ERAS strategies for analysis in the PPS. Pre-specified subgroup analyses will compare the primary and secondary outcomes across subgroups based on age, surgery type, centers, periods, compliance with ERAS strategies and so on. The results will be generated using the SAS 9.4 software package. A 2-sided P-value < 0.05 will be considered statistically significant for all other analyses.

### Data collection and management

The trial utilizes a web-based, paperless data submission system for data collection and management. All five centers participating in the study have authorized access to the data submission system. The study has defined major items for in-hospital clinical data collection, which encompass demographic characteristics, medical history, vital signs, laboratory examinations, supplementary examinations, perioperative data, complications, intervention adherence, costs and so on.The 30-day and 1-year follow-ups are conducted through telephone interviews using a standardized questionnaire. The diagnostic results are independently reviewed by two clinicians who are blinded to the randomization. In the case of any discrepancies, a consensus is reached through discussion.

### Blinding

Statisticians are blinded to the randomization schemes and treatment groups. The participants, surgeons, anesthesiologists, nurses, and other medical staff are unblinded as they will be implementing either ERAS or traditional measures for the patients.

## Discussion

Through a one-step design, prospective, multicenter cluster randomized controlled trial, we included 3030 patients planning to undergo elective congenital heart surgery under cardiopulmonary bypass in this study. Pediatric patients entering the intervention group will follow the ERAS scheme, while subjects with no intervention expectation will adhere to the current treatment routine without any intervention. By comparing the differences in the incidence of major adverse events (MACCEs, PPCs, AKI) between the two groups, we aim to compare intergroup differences in the incidence of other adverse events and length of stay. This exploration aims to determine whether the ERAS strategy can improve the outcomes of pediatric patients in compared with routines.We hypothesize that the ERAS group is superior to the control group in postoperative recovery improvement.

The application of enhanced recovery after cardiac surgery in adult is proved to be developed effectively and safely [7]. However, the development of cardiac ERAS in pediatric patients is not yet complete. There are several studies and consensus reported in the past few years. In 2019, the research team from Boston Children’s Hospital published a study, which demonstrated that ERAS can reduce mechanical ventilation time and ICU LOS relative to the pre-ERAS era [8]. In 2021, the consensus document on a comprehensive perioperative approach to enhanced recovery after pediatric cardiac surgery has been published. These perioperative measures include patient or family engagement, counseling, education, optimization, reducing fasting duration, multi-modal opioid-reducing pain management, blood conservation, normothermia, early extubation, nausea prevention, delirium prevention, delirium screening, early entreal feeding, early mobilization, non-opioid prescription, removal lines, tubes, and wires [9].

Relative studies suggested several measurements to improve pediatric outcomes. Firstly, sedation could decrease the rate of delirium and dysphoria [10, 11]. The result of studies and meta-analyses reported that the application of dexmedetomidine could reduce the accident of delirium and dysphoria, alleviate postoperative pain, and promote postoperative recovery [12, 13]. Secondly, acetaminophen and ketorolac could decrease the dose of opioid drug without increasing pain score [14–16]. Meanwhile, nerve block could be considered as an alternative analgesia method [17–19].

Studies and consensus provides valuable clinical references for ERAS implementation, however, most of the level of evidence are based on retrospective studies, which lack prospective, large-scale, and multicenter RCT investigations. Therefore, further larger sample RCT trails are needed to strengthen the level of evidence. As the concept of accelerated cardiac rehabilitation progressively expands from fast track to preoperative, intraoperative, and postoperative strategies, its clinical benefits inspire us to continually advance and enhance the speed and quality of postoperative rehabilitation for cardiac surgery pediatric patients.

### Supplementary Information

**Additional file 1: Appendix 1.** ERAC strategy.

**Additional file 2: Appendix 2.** Randomization shedule.

## Data Availability

Not applicable.
