# Supplementary material for: The protocol of Enhanced Recovery After Cardiac Surgery (ERACS) in congenital heart disease: a stepped wedge cluster randomized trial
Source: BMC Pediatr. 2024 Jan 5;24:22. doi: 10.1186/s12887-023-04422-2 (PMC10768436; doi:10.1186/s12887-023-04422-2)
Supplement: Supplementary file 1 — Additional file 1: Appendix 1. ERAC strategy. [file 12887_2023_4422_MOESM1_ESM.docx]

Appendix 1. ERAC strategy

| Phase | Content | Recommendation |
| --- | --- | --- |
| Preoperative Strategies | patient education and counseling | explanations of procedures and goals completed by surgeons, nurses, and anesthesiologists |
|  | preoperative oral intake of multidimensional carbohydrate beverages | oral administration 2 to 5 ml·kg^-1^ of energy clear beverages to pediatric patients 2 to 4 hours before surgery |
|  | sedation | parients’ company and intranasal administration of dexmedetomidine 1 to 2 μg·kg^-1^ or oral administration midazolam 0.5 mg·kg^-1^ |
| Intraoperative Strategies | continuous infusion of dexmedetomidine | inject dexmedetomidine intravenously at the rate of 0.2～0.7μg·kg^-1^·h^-1^ |
|  | multimodal analgesia | local anesthesia around the incision, or ultrasound-guided nerve block |
|  | blood conservation | tranexamic acid, cell saver, and use of modified ultrafiltration |
|  | prevention of postoperative nausea and vomiting | 0.15 mg·kg^-1^ dexamethasone or 0.1mg·kg^-1^ ondansetron before anesthesia |
| Postoperative Strategies | early extubation | extubation immediately after surgery or within 6 hours of surgery |
|  | multimodal analgesia | oral application of acetaminophen 4 times daily with a total daily dose of 30 mg/kg, ketorolac would be recommended to injected intravenously as needed, with a single dose of 0.5 to 1 mg·kg^-1^. |
|  | goal-directed fluid therapy | goals (blood pressure, cardiac index, systemic venous oxygen saturation, and so on) to guide clinicians in administering fluids, vasopressors, and inotropes |
|  | early feeding and physical exercise | drinking can be attempted 2 hours after extubation and sitting or exercising can be attempted 4 hours after extubation |
