# Supplementary material for: The protocol of Enhanced Recovery After Cardiac Surgery (ERACS) in congenital heart disease: a stepped wedge cluster randomized trial
Source: BMC Pediatr. 2024 Jan 5;24:22. doi: 10.1186/s12887-023-04422-2 (PMC10768436; doi:10.1186/s12887-023-04422-2)
Supplement: Supplementary file 2 — Additional file 2: Appendix 2. Randomization shedule. [file 12887_2023_4422_MOESM2_ESM.docx]

Appendix 2. Randomization shedule

| Sequence | Site | T1 | T2 | T3 | T4 | T5 | T6 |
| --- | --- | --- | --- | --- | --- | --- | --- |
| 1 | 1 | C | I | I | I | I | I |
| 2 | 2 | C | C | I | I | I | I |
| 3 | 3 | C | C | C | I | I | I |
| 4 | 4 | C | C | C | C | I | I |
| 5 | 5 | C | C | C | C | C | I |

Randomization schedule consisting of 5 sequences and 6 periods(T1 to T6) for the 5 participating study sites. T1: Months 1 to 2, T2: Months 3 to 4, T3: Months 5 to 6, T4: Months 7 to 8, T5:Months 9 to 10, T6: Months 11 to 12.
